# Complete mitochondrial genome of the phenotypically-diverse sea urchin *Strongylocentrotus intermedius* (Strongylocentrotidae, Echinoidea)

**DOI:** 10.1080/23802359.2017.1372727

**Published:** 2017-09-04

**Authors:** Evgeniy S. Balakirev, Vladimir A. Pavlyuchkov, Francisco J. Ayala

**Affiliations:** a Department of Ecology and Evolutionary Biology, University of California, Irvine, CA, USA;; b A.V. Zhirmunsky Institute of Marine Biology, National Scientific Center of Marine Biology, Far Eastern Branch, Russian Academy of Sciences, Vladivostok, Russia;; c School of Natural Sciences, Far Eastern Federal University, Vladivostok, Russia;; d Pacific Research Fisheries Centre (TINRO-Centre), Vladivostok, Russia

**Keywords:** Sea urchins, *Strongylocentrotus intermedius*, morphological variability, incipient species, mitochondrial genome

## Abstract

The complete mitochondrial genomes are sequenced in two individuals representing two morphological forms, ‘usual’ (U) and ‘gray’ (G), of the short-spined sea urchin *Strongylocentrotus intermedius*. The genome sequences are 15,705 bp in size, and the gene arrangement, composition, and size are very similar to the other sea urchin mitochondrial genomes published previously. A low level of sequence divergence (*D*_xy_ = 0.0083 ± 0.0007) is detected between the forms. The GenBank (KC490912) mt genome of *S. intermedius* is much closer to the U form (*D*_xy_ = 0.0013 ± 0.0003) than to the G form (*D*_xy_ = 0.0085 ± 0.0006), demonstrating unique evolutionary trajectories for each form, which we previously suggested based on the *bindin* gene and symbiont analyses.

The short-spined sea urchin *Strongylocentrotus intermedius* (A. Agassiz) inhabits a wide range of the northwest Pacific region, including the Sea of Japan and the Sea of Okhotsk (Jensen 1974; Bazhin and Stepanov 2012). There are two sympatric morphological forms, ‘usual’ (U) and ‘gray’ (G), which are different in morphology and preferred bathymetric distribution. We detected previously that these two forms harbour highly divergent bacterial symbiont lineages and show distinct patterns of nuclear *bindin* gene variability but not mitochondrial *COI* gene (Balakirev et al. 2008, 2016). The evolutionary history and taxonomical relationships of the *S. intermedius* morphological forms remain unclear due to limited genetic data.

We have sequenced two complete mitochondrial (mt) genomes of *S. intermedius* represented by two morphological forms (GenBank accession numbers KY964299, KY964300) from a single sea urchin settlement within a distance of ∼150 m (46°15.086′ N, 138°06.646′ E; Cape Zolotoi, Sea of Japan). The U and G forms were collected at depths of 5–10 m and 15–25 m, respectively. The mt fragments were amplified with primers designed with the program mitoPrimer, v. 1 (Yang et al. 2011). The tissue samples are stored at the A. V. Zhirmunsky Institute of Marine Biology museum (Vladivostok, Russia); accession numbers MIMB 33945 and MIMB 33946.

The genome sequences of *S. intermedius* are 15,705 bp in size, and the gene arrangement, composition, and size are very similar to other strongylocentrotid sea urchin mitochondrial genomes published previously. There are 131 single nucleotide and one length (within the *ND6* gene) differences between the two haplotypes (SIT17 and SIG19); total sequence divergence (*D*_xy_) is 0.0083 ± 0.0007. Comparison of the new mt genomes with mt genomes available in GenBank for the genera *Strongylocentrotus*, *Mesocentrotus*, and *Pseudocentrotus* reveals a close affinity of *S. intermedius* to other strongylocentrotid sea urchins (Figure 1) with low divergence (*D*_xy_ = 0.0049 ± 0.0004) between our specimens and the GenBank *S. intermedius* (KC490912) complete mt genome.

**Figure 1.**
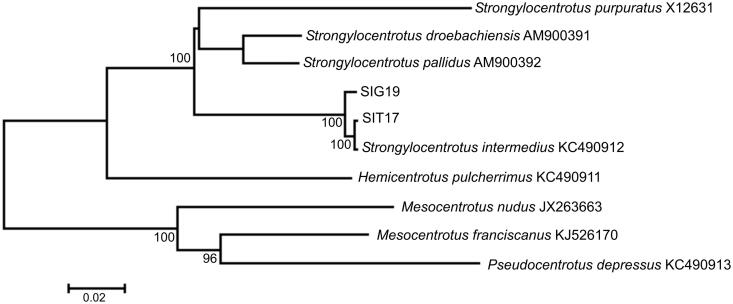
Maximum likelihood tree for the short-spined sea urchin *Strongylocentrotus intermedius* specimens SIG19 and SIT17, and GenBank representatives of the class Echinoidea. The tree is constructed using whole mitochondrial genomes. The tree is based on the general time reversible + gamma + invariant sites (GTR + G + I) model of nucleotide substitution. The numbers at the nodes are bootstrap percent probability values based on 500 replications (values below 75% are omitted).

The GenBank *S. intermedius* specimen (KC490912) was collected from a depth less than 10 m in Jumunjin Harbor, Sea of Japan, South Korea (Dr. Youn-Ho Lee, Korea Institute of Ocean Science and Technology) and represented the U form inhabiting shallow-water settlements (5–10 m). The Jumunjin Harbor is ∼2089.43 km along the coastline from Cape Zolotoi, where we collected our specimens. Despite the long distance, the nucleotide difference between the GenBank and the U form is low (*D*_xy_ = 0.0013 ± 0.0003), but 6.5 times higher (*D*_xy_ = 0.0085 ± 0.0006) between the GenBank specimen and the G form. Thus, our specimens, collected in close proximity (∼150 m) but belonging to different morphological forms, are more different than specimens collected from distant regions (separated by ∼2089.43 km) but belonging to the same morphological form. These discrepancies between genetic differences and geographical distances confirm that the U and G morphological forms of *S. intermedius* represent distinct evolutionary lineages (incipient species) with low genetic divergence but significant differences in symbiont contents (Balakirev et al. 2008, 2016).
